# Myxoma Virus Expressing a Fusion Protein of Interleukin-15 (IL15) and IL15 Receptor Alpha Has Enhanced Antitumor Activity

**DOI:** 10.1371/journal.pone.0109801

**Published:** 2014-10-16

**Authors:** Vesna Tosic, Diana L. Thomas, David M. Kranz, Jia Liu, Grant McFadden, Joanna L. Shisler, Amy L. MacNeill, Edward J. Roy

**Affiliations:** 1 Department of Molecular and Integrative Physiology, University of Illinois at Urbana-Champaign, Urbana, Illinois, United States of America; 2 Neuroscience Program, University of Illinois at Urbana-Champaign, Urbana, Illinois, United States of America; 3 Department of Biochemistry, University of Illinois at Urbana-Champaign, Urbana, Illinois, United States of America; 4 Department of Molecular Genetics and Microbiology, University of Florida, Gainesville, Florida, United States of America; 5 Department of Microbiology, University of Illinois at Urbana-Champaign, Urbana, Illinois, United States of America; 6 Department of Pathobiology at College of Veterinary Medicine, University of Illinois at Urbana-Champaign, Urbana, Illinois, United States of America; Swedish Medical Center, United States of America

## Abstract

Myxoma virus, a rabbit poxvirus, can efficiently infect various types of mouse and human cancer cells. It is a strict rabbit-specific pathogen, and is thought to be safe as a therapeutic agent in all non-rabbit hosts tested including mice and humans. Interleukin-15 (IL15) is an immuno-modulatory cytokine with significant potential for stimulating anti-tumor T lymphocytes and NK cells. Co-expression of IL15 with the α subunit of IL15 receptor (IL15Rα) greatly enhances IL15 stability and bioavailability. Therefore, we engineered a new recombinant myxoma virus (vMyx-IL15Rα-tdTr), which expresses an IL15Rα-IL15 fusion protein plus tdTomato red fluorescent reporter protein. Permissive rabbit kidney epithelial (RK-13) cells infected with vMyx-IL15Rα-tdTr expressed and secreted the IL15Rα-IL15 fusion protein. Functional activity was confirmed by demonstrating that the secreted fusion protein stimulated proliferation of cytokine-dependent CTLL-2 cells. Multi-step growth curves showed that murine melanoma (B16-F10 and B16.SIY) cell lines were permissive to vMyx-IL15Rα-tdTr infection. *In vivo* experiments in RAG1^-/-^ mice showed that subcutaneous B16-F10 tumors treated with vMyx-IL15Rα-tdTr exhibited attenuated tumor growth and a significant survival benefit for the treated group compared to the PBS control and the control viruses (vMyx-IL15-tdTr and vMyx-tdTr). Immunohistological analysis of the subcutaneous tumors showed dramatically increased infiltration of NK cells in vMyx-IL15Rα-tdTr treated tumors compared to the controls. *In vivo* experiments with immunocompetent C57BL/6 mice revealed a strong infiltrate of both NK cells and CD8^+^ T cells in response to vMyx-IL15Rα-tdTr, and prolonged survival. We conclude that delivery of IL15Rα-IL15 in a myxoma virus vector stimulates both innate and adaptive components of the immune system.

## Introduction

The oncolytic potential of many viruses, such as the poxviruses vaccinia virus and myxoma virus, initially suggested that they could be used as cancer therapy, but the efficacy of such viruses as a single agent *in vivo* has been limited [1]. Alternatively, the selectivity of such oncolytic and oncotropic viruses can be used to deliver cytokine genes to cancer cells [2],[3]. One goal of this approach is to shift the immunosuppressive microenvironment found in many solid tumors to an environment that better favors the induction of anti-tumor immune responses.

Myxoma virus is an oncotropic poxvirus that has a particularly attractive safety profile. In the wild, the virus infects only rabbits and other related leporids, and is nonpathogenic in all other nonlagomorph animals tested [4]. Despite its lack of broad pathogenicity other than the rabbit, myxoma virus can replicate in diverse cultured cells from many species, including most human cancer cells, which are particularly permissive for the virus [5],[6],[7]. It also selectively infects tumors in human xenograft models [8],[9],[10],[11] and primary mouse tumors [12],[13],[11]. It has recently been shown that myxoma virus can discriminate cancerous human myeloid cells from normal CD34^+^ stem cells, which makes it a potential *ex vivo* purging agent for hematological malignancies [14],[15].

Some oncotropic viruses tested in clinical trials have been modified to express an immunostimulatory cytokine, GM-CSF [16],[17]. Although GM-CSF is a cytokine with potentially favorable anti-tumor activity, it can also stimulate suppressive components of the immune system [18]. Therefore, it is worth exploring other cytokine candidates to be delivered by a tumor-selective viral vector, particularly those that are known to be capable of activating non-responsive or anergic cytotoxic lymphocytes [19].

IL15 is a pro-inflammatory cytokine with significant potential for stimulating T lymphocytes and NK cells against cancer [20]. IL15 expression is tightly regulated at the post-transcriptional level, making IL15 protein largely detectable only in monocytes/macrophages and dendritic cells [21]. Co-expression of IL15 with the α subunit of IL15 receptor (IL15Rα) greatly enhances IL15 stability and function *in vivo* [22], [23],[24]. Since IL-15Rα may be considered a part of the active IL-15 cytokine complex rather than part of the receptor, pre-association of IL-15 with IL-15Rα generates a more potent ligand compared to the cytokine alone [25],[26],[27],[28]. Recombinant myxoma viruses have previously been engineered to express tdTomato red fluorescent protein (vMyx-tdTr) and mouse interleukin-15 (vMyx-IL15-tdTr) [29]. Our previous studies have shown that these myxoma viruses (vMyx-tdTr and vMyx-IL15-tdTr) productively infect cancer cells *in vitro*, but have limited effect on tumor progression of murine melanoma in immune competent mice *in vivo* [30],[31]. In order to deliver the biologically potent form of IL15 with its IL15Rα component *in vivo*, we engineered a new recombinant myxoma virus (vMyx-IL15Rα-tdTr), which expresses IL15Rα-IL15 fusion protein, as well as tdTomato red fluorescent reporter protein.

In this study, we describe the therapeutic effects observed with the new recombinant virus in a mouse model of aggressive melanoma, B16-F10. *In vitro* testing of the virus showed that B16-F10 cells are permissive to the vMyx-IL15Rα-tdTr infection. Secretion of the IL15Rα-IL15 fusion protein was confirmed by ELISA and functional activity of the fusion was assessed by a proliferation assay on IL15-dependent CTLL-2 cells. In *in vivo* experiments, immunohistological analysis of the subcutaneous tumors showed dramatically increased infiltration of NK cells in vMyx-IL15Rα-tdTr treated tumors compared to controls in RAG1^-/-^ mice. In immunocompetent C57BL/6 mice, vMyx-IL15Rα-tdTr increased infiltration by NK cells and CD8^+^ T cells. RAG1^-/-^ mice with subcutaneous B16-F10 tumors were treated with vMyx-IL15Rα-tdTr, resulting in a significant survival benefit for the treated group compared to the PBS control and the control viruses (vMyx-IL15-tdTr that expresses the native IL15 ligand and control vMyx-tdTr). Treatment of tumor-bearing C57BL/6 mice with vMyx-IL15Rα-tdTr resulted in longer survival than similarly treated RAG1^-/-^ mice. Our results suggest that virally delivered IL15Rα-IL15 drives the recruitment of NK cells and T cells to the site of the tumor and that both the innate and adaptive components of the host immune system play a role in the anti-tumor effect.

## Materials and Methods

### DNA constructs

pBluescript SK+ plasmid that served as a cloning backbone for the IL15Rα-IL15 – tdTomato expression cassette was obtained from ATCC (Manassas, VA). Whole viral DNA isolated from vMyx-tdTr [29] using the DNeasy Tissue Kit (Qiagen, Valencia, CA) was used as a template for obtaining the PCR fragment containing partial sequences of M135 and M136 genes. HindIII and BamHI cutting sites were introduced in this PCR reaction; primers for all PCR reactions were obtained from Integrated DNA Technologies (Coralville, IA) (Forward primer: 5′- CCA AAG CTT CAC CTG TGT ATG TT -3′, Reverse primer: 5′- CCA GGA TCC ATA ACA CAC AGT TCG G -3′). PCR product from vMyx-tdTr was ligated into pBluescript using the T4 Ligase (Invitrogen, Carlsbad, CA) following sequential digestion with HindIII and BamHI (New England BioLabs, Ipswich, MA). IL15Rα-IL15 fusion protein contains codon-optimized sequence for the murine IL-15Rα sushi domain (amino acids 34–103 of Isoform 1, UniProt accession #Q60819), a linker with the sequence GG(SGG)_6_ and murine IL-15; it was purchased from GenScript (Piscataway, NJ) [32]. Poxvirus vvSynE/L promoter, murine Ig κ-chain leader sequence (which directs the protein to the secretory pathway) as well as BspEI and NdeI cutting sites were added and His tag was eliminated from the original IL15Rα-IL15 sequence using forward primer: 5′- CGC AGC TCC GGA AAA AAT TGA AAT TTT ATT TTT TTT TTT TGG AAT ATA AAT AAG ATG GAG ACA GAC ACA CTC CTG CTA TGG GTA CTG CTG CTC TGG GTT CCA GGT TCC ACT GGT GAC ACC ACC TGC CCC CCC CCC GTG -3′ and reverse primer: 5′- TCG CGC CAT ATG TTA TCA GCT GGT GTT GAT GAA CAT CTG CAC G -3′. The resulting IL15Rα-IL15 sequence was cloned into the earlier described pBluescipt construct using BspEI and NdeI (New England BioLabs, Ipswich, MA) and T4 Ligase (Invitrogen, Carlsbad, CA). Finally, tdTomato was cloned out of the plasmid provided by Dr. Brian Freeman (University of Illinois) using primers: forward 5′- GCA GTC GAC ATG GTG AGC AAG G - 3′ and reverse: 5′- CCT GAA TTC TTA CTT GTA CAG CTC G - 3′ and cloned into the existing pBluescript construct using SalI and EcoRI. Resulting plasmid, pBS-IL15Rα-IL15-tdTomatoRed (Figure S1) was used for creating vMyx-IL15Rα-tdTr.

### Recombinant viruses

The Lausanne strain of myxoma virus (vMyx-Lau) was used to create recombinant virus expressing tandem dimer Tomato red fluorescent protein (vMyx-tdTr) with or without expression of interleukin-15 (vMyx-IL15-tdTr) by intergenic insertion of the gene cassettes between M135R and M136R of the myxoma virus genome as previously described [29]. Protein expression of IL15 by this recombinant virus is driven by a vaccinia virus late promoter (p11).

The recombinant virus expressing the IL15Rα-IL15 fusion protein (vMyx-IL15Rα-tdTr) was created by homologous recombination in RK-13 cells infected with wild type (WT) vMyx-Lau followed by transfection with the engineered recombination vector pBS-IL15Rα-IL15-tdTomatoRed (Figure S1). The recombination vector contains genes for the IL15Rα-IL15 fusion protein and tdTomato, both under control of the same synthetic vaccinia virus early/late promoter (vvSynE/L promoter). This expression cassette is flanked by M135 and M136 partial gene sequences for the purpose of being transfected into the WT myxoma virus genome between genes M135 and M136. Myxoma virus permissive RK-13 cells were infected with WT vMyx-Lau followed by cationic lipid transfection of the engineered recombination vector pBS with the IL15Rα-IL15-tdTr cassette. After plasmid recombination into the virus, recombinant virus expressing IL15Rα fusion protein was propagated and titrated by focus formation on RK-13 cells. Fluorescent virus foci were harvested, repropagated and titrated on RK-13 cells. This process was repeated three times to isolate a purified virus which contains two exogenous genes; IL-15Rα-IL15 fusion protein and tdTomato. Genomic structure of recombinant virus was confirmed by PCR sequencing.

### Cell culture and reagents

Rabbit kidney epithelial (RK-13) cells were a gift from Dr. Richard Moyer (University of Florida, Gainesville, FL; originally from ATCC, Manassas, VA). RK-13 are grown at 37°C, 5% CO2, and 100% humidity in minimum essential medium with Earle's salts (Mediatech, Manassas, VA) supplemented with 2 mM glutamine, 50 U/mL penicillin G, 50 µg/mL streptomycin, 1 mM sodium pyruvate, 0.1 mM nonessential amino acids (MEM-C), and 10% fetal bovine serum (FBS; HyClone, Logan, UT).

The murine melanoma cell line, B16-F10 was purchased from ATCC (Manassas, VA). The murine glioma cell line GL261 was obtained from the National Cancer Institute-Frederick Cancer Research Tumor Repository (Frederick, MD). B16.SIY was derived from B16-F10 cells retrovirally transduced to express green fluorescent protein (GFP) as a fusion protein with SIYRYYGL (SIY) [33], [34] and was a gift from Dr. Thomas Gajewski (University of Chicago, Chicago, IL).

Cancer cell lines were cultured in complete Roswell Park Memorial Institute (RPMI) 1640 medium containing 5 mM HEPES, 1.3 mM L-glutamine, 50 µM 2-ME, penicillin, streptomycin and 10% fetal bovine serum (FBS) at 37°C and 5% CO_2_.

Cytotoxic T Lymphocyte Line 2 (CTLL-2) cytokine-dependent murine T cell line (ATCC, Manassas, VA) was cultured in complete RPMI 1640 medium additionally supplemented with 10% T-Stim (culture supernatant from rat T cells stimulated with ConA from BD Biosciences, San Jose, CA).

### Viral growth curves

Tested cell lines were plated into 6-well cell culture plates (Nunc, Roskilde, Denmark) and grown in MEM-C with 10% FBS until they reached 90–95% confluency. For multi-step growth curves cells were inoculated with vMyx-IL15Rα-tdTr or vMyx-tdTr diluted in 400 µL MEM-C at a multiplicity of infection (MOI) of 0.1 plaque-forming units (PFU) per cell. Inoculated cells were incubated at 37°C and 5% CO_2_ for 1 h, rocking every 15 min. Next, virus was removed, cell monolayers were washed with phosphate-buffered saline (PBS), and MEM-C with 10% FBS was added to each well. Inoculations of each cell line were performed in triplicate. At 0, 4, 8, 12, 24 and 48 h post-inoculation, cells were dislodged by scraping into media. Cells were collected by centrifugation at 500xg for 5 min. Next, each supernatant was removed, and the cellular pellet was resuspended in 0.5 ml PBS and stored at −80°C. Prior to titering the virus, cells were disrupted by three freeze/thaw cycles and sonication in order to release the virus from the cells. Samples were titered in duplicate. Titering was performed by plating 10-fold serial dilutions of the samples in MEM-C onto RK-13 monolayers in 6-well or 24-well culture plates. The inoculated cells were incubated for 1 h at 37°C and 5% CO_2_, then the inoculum was removed and an overlay consisting of equal amounts of 1% agarose (Lonza, Rockland, ME) and 2×MEM-C with 20% FBS was added on RK-13 cells. Viral plaques were visualized as small white foci (red foci under fluorescent light) and counted at 6-7 days post-inoculation (dpi).

### ELISA analysis of the IL15Rα-IL15 fusion protein

RK-13 cells were plated in 6-well culture plates and upon reaching 90–95% confluency they were inoculated with vMyx-IL15Rα-tdTr or vMyx-tdTr diluted in 400 µL MEM-C at MOI of 5 PFU/cell. After 1 h incubation at 37°C and 5% CO_2_, inoculum was replaced with MEM-C with 10% FBS. At different time points post-inoculation, both cell supernatant and cellular extract were collected. Supernatants were centrifuged briefly to remove cellular debris and clarified supernatants were transferred to new tubes and stored at −80C. The remaining cellular monolayer was detached from the well by scraping cells into 1 ml PBS. Cells were collected, pelleted by brief centrifugation (1,300 rpm x 1 min), and cellular pellets were resuspended in Cytoplasmic Extract (CE) buffer supplemented with HALT protease inhibitor cocktail (Thermo Fisher, Rockford, IL). Samples were incubated for 5 min at 4°C and centrifuged at 1,300 rpm for 1 min. Supernatants were moved to new tubes are stored at −80°C.

For IL15Rα-IL15 detection by ELISA, the Mouse IL-15/IL-15R Complex ELISA Ready-SET-Go! kit (eBioscience, San Diego, CA) was used. All samples were 10-fold serially diluted and each dilution was done in duplicate. Each kit included a purified protein standard which was used to establish a standard curve. An ELx800 Absorbance Microplate Reader (BioTek Instruments, Winooski, VT) was used to detect absorbance at 450 nm.

### CTLL-2 cell proliferation assay

MTT reagent (3-(4,5-dimethylthiazolyl-2)-2,5-diphenyltetrazolium bromide) and detergent were purchased from ATCC (Manassas, VA). Confluent RK-13 cells in 6-well plates were infected with vMyx-IL15Rα-tdTr or vMyx-tdTr at MOI = 5. At 24 h and 48 h p.i., cell-free media was collected and stored at −80°C. CTLL-2 cells were propagated overnight at 37°C, 5% CO_2_ in complete RPMI media with no added cytokines. Next, CTLL-2 cells were collected by centrifugation and resuspended at 50,000 cells per well in a 96-well plate in 100 µL complete RPMI containing either 10^−9^M IL-2, 10^−9^M TCR-IL15Rα (fusion of the m33 TCR with IL15Rα-IL15, “m33-superfusion” [32]), vMyx-tdTr or vMyx-IL15Rα-tdTr infected cell media. The cells were cultured for 48 h, and then 10 µL MTT was added per well, and the cells were incubated at 37°C, 5% CO_2_ for three more hours, and then 100 µL per well detergent was added, and the plate was incubated at room temperature overnight. To estimate CTLL-2 cell proliferation in different conditions, absorbance at 570 nm in each well was read using an EL_x_800 universal microplate reader (Bio-Tek Instruments, Winooski, VT).

### Animals

C57BL/6 and C57BL/6 RAG1^-/-^ mice originally purchased from The Jackson Laboratory (Bar Harbor, ME, USA) were maintained as colonies and housed in the animal facilities at the University of Illinois. Mice were used in experiments when they were 2–5 months old. All animal studies were approved by the Institutional Animal Care and Use Committee at the University of Illinois Urbana-Champaign (PHS Assurance A3118-01, AAALAC, International Accreditation #00766). Anesthesia was used during tumor cell and virus injections, and all efforts were made to minimize suffering.

### Subcutaneous tumor establishment and treatment

B16-F10 melanoma cells were harvested and washed twice with Hanks Balanced Salt Solution (HBSS, Cellgro Mediatech Inc., Manassas, VA). Prior to all procedures, mice were anesthetized by isoflurane (Aerrane, Baxter, Deerfield, IL) inhalation using the classic vaporizer unit by E–Z Systems (Palmer, PA). Shaved mice received 1x 10^6^ tumor cells in 100 µl HBSS subcutaneously into the right flank. After 7 days, when tumors usually reach a volume of approximately 100 mm^3^, mice were assigned to treatment groups and received an intratumoral (i.t.) injection of virus. At this time, tumors were directly injected with 2.6×10^7^ PFU of sucrose-pad purified vMyx-IL15α-tdTr, vMyx-IL-15-tdTr or vMyx-tdTr that was in a final volume of 50 µl. A separate set of mice received 50 ul PBS i.t. An additional i.t. inoculation of each virus (2.6×10^7^ PFU) occurred 3 days later (day 10 post-implantation). For those tumors that were large, the inoculum was injected into at least three different sites to introduce the virus throughout the mass. Prior to all repeated tumor injections with either virus or PBS, animals were anesthetized by isoflurane inhalation as described earlier. All animals were single housed upon tumor cell implantation and during all subsequent experimental manipulations.

Tumor growth was monitored by measuring tumor length, width and height with a caliper. Tumor volume was calculated as ((length) x (width) x (height))/2. Mice were monitored daily. Mice were humanely euthanized when tumors reached the volume of 3000 mm^3^, or showed lethargy or signs of pain, or when reaching 75% baseline body weight. Mice were euthanized by CO_2_ asphyxiation followed by cervical dislocation. In some experiments, samples from some mice were collected 3 days after final virus treatment for histological analysis, and other mice in each treatment group were monitored until they reached a criterion for euthanasia.

### Tissue sections and immunostaining

After the mice were euthanized, their subcutaneous tumors were snap-frozen in OCT medium for cryosectioning and immunostaining. Eight µm cryosections were taken. Primary antibodies used for staining were: 4D11 (rat anti-Ly-49G2, BD Pharmingen, San Jose, CA), rabbit anti-CD3 (Abcam, Cambridge, MA), rat anti-CD8 (eBioscience, San Diego, CA), rat anti-CD4 (Abcam, Cambridge, MA). Secondary antibodies used: biotin rabbit anti-rat and biotin goat anti-rabbit (Vector, Burlingame, CA). For immunostaining, slides were fixed in cold 95% ethanol and blocked with Superblock (Thermo Scientific, Rockford IL). Sections were then incubated with a primary antibody in PBS+20% glycerol (PBSG) overnight, washed with PBS+0.1% Tween-20 (PBST), and incubated with biotinylated secondary anibody in PBST for 4 h. Slides were washed and incubated with streptavidin-Alexa Fluor 594 (Invitrogen, Carlsbad, CA) or streptavidin-Alexa Fluor 488 (Jackson ImmunoResearch, West Grove, PA) and DAPI (Invitrogen, Carlsbad, CA). Control slides omitting the primary antibody were negative for Alexa Fluor 594 or Alexa Fluor 488. Images were obtained with an Olympus BX-51 microscope at 20x magnification.

### Data Analysis

GraphPad Prism software (La Jolla, CA) was used for all statistical analyses and graph presentation. Survival data were recorded from the time of the tumor cell implantation until euthanasia and were plotted using a Kaplan-Meier curve. Survival treatment groups were compared with a Log-rank (Mantel-Cox) test. Virus growth curves were analyzed by two-way ANOVA, ELISA data were analyzed by t-test at corresponding time points, the bioassay of IL-15 activity was analyzed by one-way ANOVA, and histological cell counts were analyzed by one-way ANOVA with Bonferroni planned comparisons. Significance was considered p<0.05.

## Results

### Murine melanoma and glioma cell lines are permissive for recombinant myxoma virus infection

**Figure 1:**
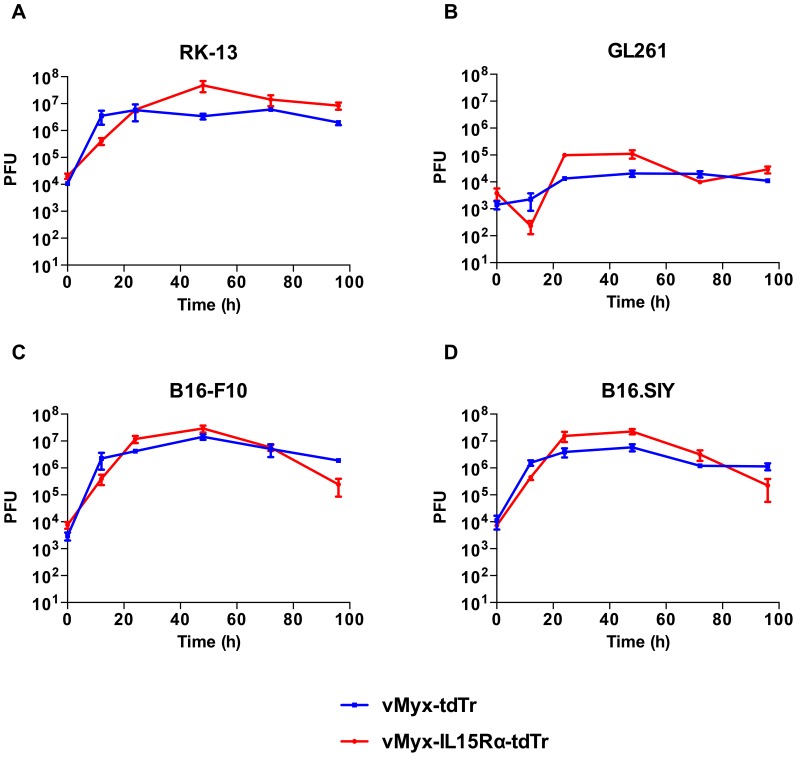
Melanoma and glioma cell lines are permissive to recombinant myxoma virus infection. Cell lines (A) RK-13, (B) GL261, (C) B16-F10, (D) B16.SIY, were infected with either vMyx-tdTr or vMyx-IL15Rα-tdTr at a multiplicity of infection (MOI) of 0.1 to obtain multi-step growth curves. At 0, 12, 24, 48, 72 or 96 h post-infection (p.i.), cells were harvested and lysed, and the viral titer was determined by titration on RK-13 cells. Error bars represent SEM from 3 replicates for each cell line. There was a significant effect of time for each of the cell lines (p<0.001).

Prior to testing the therapeutic capacity of the recombinant IL15Rα-IL15 virus (vMyx-IL15Rα-tdTr), we tested its capacity to infect relevant murine cancer cell lines *in vitro*. Multi-step growth curves of vMyx-IL15Rα-tdTr and the previously characterized vMyx-tdTr control virus [29], showed similar patterns of permissiveness in various cell lines that were tested (Figure 1). Melanoma cell lines (B16-F10 and B16.SIY) were as permissive as the positive control rabbit cell line RK-13. For all three cell lines, infectious viral particles were formed by 12 h post infection, and maximal viral titer was typically obtained at the 48 h time point. vMyx-IL15Rα-tdTr and vMyx-tdTr showed a different growth phenotype in the glioma cell line (GL261) and produced lower viral titers (Figure 1B). Based on the observation that the insertion of the IL15Rα-IL15 gene did not impact the infectivity of the virus, vMyx-IL15Rα-tdTr was considered a useful system to deliver functional IL15Rα-IL15 fusion protein to B16 tumors.

### IL15Rα-IL15 fusion protein is expressed and secreted *in vitro* by virus infected cells

**Figure 2:**
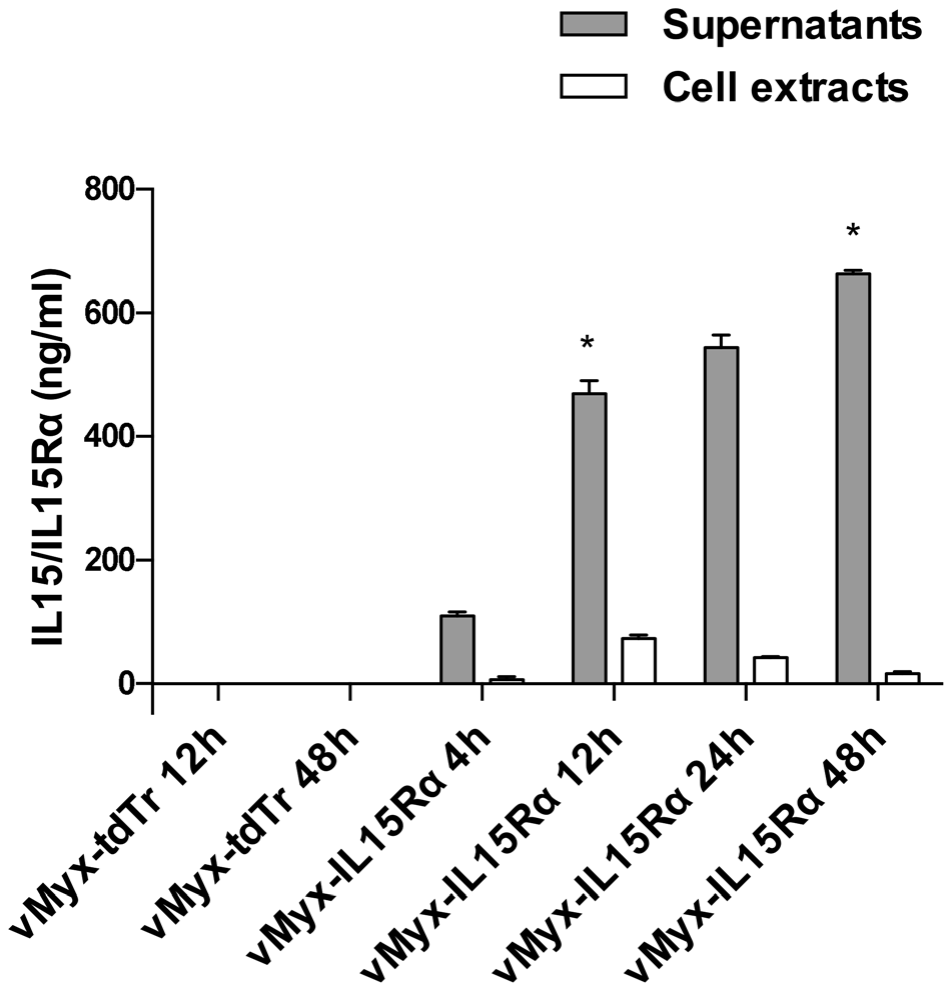
IL15Rα-IL15 fusion protein is present in the supernatants and extracts of cells infected with vMyx-IL15Rα-tdTr. Confluent RK-13 cells in 6-well plates were infected with vMyx-IL15Rα-tdTr or vMyx-tdTr at MOI = 5. At indicated times post-infection, media was collected and cells were scraped, lysed and cytoplasmic extract was harvested. Mean ELISA values with SEM for replicates of the same condition are presented, and the experiment was repeated with similar results. There was a significant increase in IL15Rα-IL15 fusion protein in supernatants of vMyx-IL15Rα-tdTr treated cells compared to vMyx-tdTr treated ones at corresponding timepoints (* - p<0.05).

To determine if cells infected with vMyx-IL15Rα-tdTr were capable of secreting IL15Rα-IL15 fusion protein, we examined the culture media and cell extracts of infected cells using an ELISA specific for the IL15/IL15R complex. IL15Rα-IL15 was detected in both supernatants and cell extracts of vMyx-IL15Rα-tdTr infected RK-13 cells (MOI = 5) as compared to the control non-cytokine expressing virus vMyx-tdTr (Figure 2). The peak of cell-associated expression of the fusion protein occurred at 12 h post-infection (mean value of 73 ng/ml), while secreted levels peaked at 48 h post-infection (mean value of 663 ng/ml). IL15Rα-IL15 was present in ten-fold higher levels in cellular supernatants versus cell-associated. As would be expected, cells infected with non-cytokine expressing virus (vMyx-tdTr) did not show measurable levels of IL15Rα-IL15. The presence of IL15 and IL15Rα domains was also confirmed by Western blot of supernatants and cell extracts (Figure S2 and Materials and Methods S1). These findings showed that the recombinant virus was transcribed effectively and that the translated IL15Rα-IL15 fusion protein was secreted from infected cells at high levels (over 500 ng/ml).

### IL15Rα-IL15 fusion protein secreted by vMyx-IL15Rα-tdTr infected cells is functionally active

**Figure 3:**
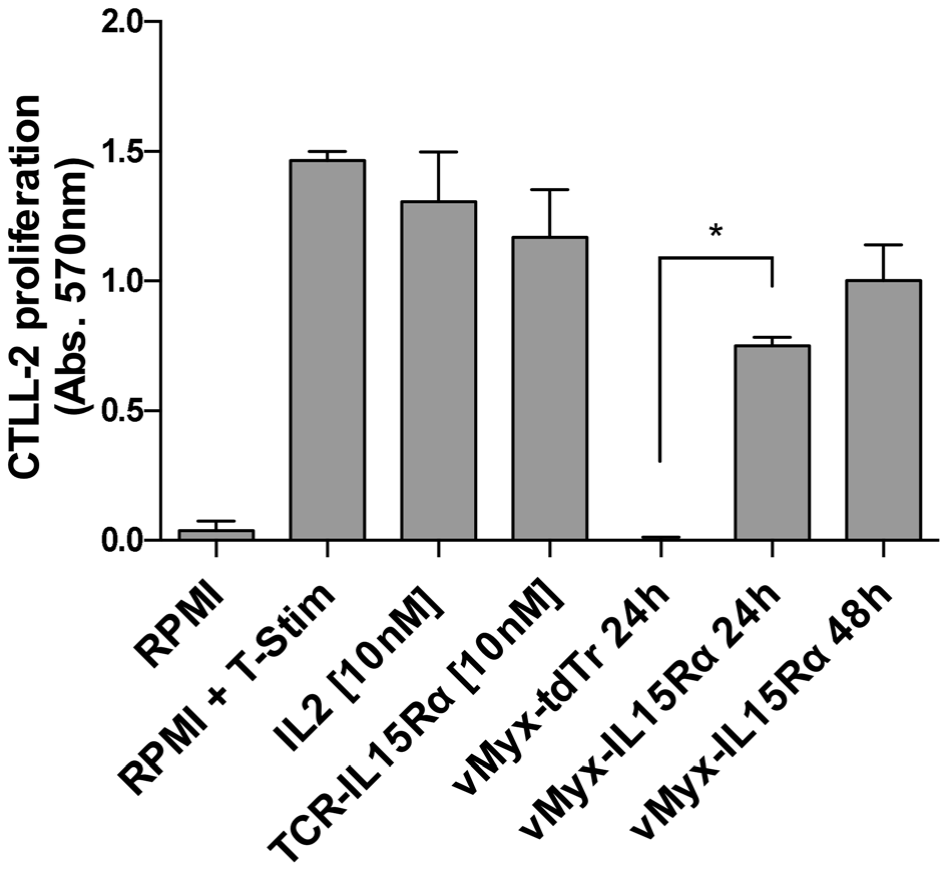
IL15Rα-IL15 fusion protein secreted by virus-infected RK-13 cells is functionally active. Confluent RK-13 cells in 6-well plates were infected with vMyx-IL15Rα-tdTr or vMyx-tdTr at MOI = 5. At 24 h and 48 h p.i., cell media was collected. Cytokine dependent CTLL-2 cells were incubated with un-supplemented media (RPMI), three positive controls (media supplemented with 10% T-Stim (RPMI + T-Stim), 10^-9^M IL-2, 10^-9^M TCR-IL15-IL15Rα fusion protein (TCR-IL15Rα)), and vMyx-tdTr or vMyx-IL15Rα-tdTr supernatants. CTLL-2 cell proliferation was analyzed by the MTT assay. Experiment was done in triplicate for each treatment, and mean values with SEM are presented. Positive controls and supernatants from RK-13 cells infected with vMyx-IL15Rα-tdTr (marked by *) showed significant functional IL-15 activity, but supernatant from vMyx-tdTr infected RK-13 cells did not.

Functional activity of the IL15Rα-IL15 fusion protein in the supernatants of virus-infected cells was assayed by its ability to induce proliferation of cytokine-dependent CTLL-2 cells (Figure 3). CTLL-2 is a clone of T cells that requires IL-2 or other growth-promoting cytokines for proliferation [35]. MTT cell proliferation assays showed that CTLL-2 cells cultured in medium supplemented with supernatants of vMyx-IL15Rα-tdTr infected cells proliferated to the similar extent as CTLL-2 cells incubated with recombinant IL-2 (10 nM) or a purified fusion protein of IL15Rα-IL15 and a single-chain TCR m33 (10 nM) [32]. CTLL-2 cells cultured with supernatant of the control virus vMyx-tdTr were not stimulated to proliferate.

### Treatment with IL15Rα-IL15 fusion protein expressing myxoma virus results in increased presence of NK cells in tumors of RAG1^-/-^ mice

**Figure 4:**
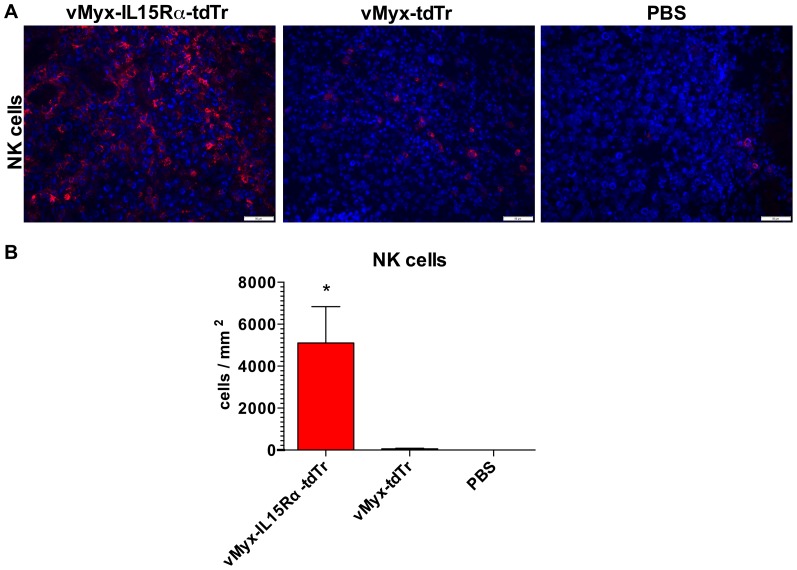
NK cell infiltration of subcutaneous B16-F10 tumors 3 days after intratumoral virus treatment in RAG1^-/-^ mice. RAG1^-/-^ mice (n = 3 per group) were implanted with unilateral subcutaneous B16-F10 tumor cells. The first dose of the virus (2.6×10^7^ PFU i.t.) was given on day 7 (when tumors reached approximately 100 mm^3^) and the second dose was given on day 10. Treatment groups are: 1. vMyx-IL15Rα-tdTr 2. vMyx-tdTr 3. PBS. Mice were euthanized 3 days after the final virus treatment and tumor sections were analyzed for presence of NK cells by immunostaining for Ly-49G2 (4D11 antibody). Representative tumor sections are shown. (A) Staining for NK cells in tumors. Red – 4D11-positive stain, Blue – DAPI. Scale bar  = 50 micrometers. (B) Estimated number of NK cells per square millimeter of a tumor section for each condition. Presented values are mean cell count in tumors from three mice, with SEM. One-way ANOVA showed significant increase in NK cell accumulation in vMyx-IL15Rα-tdTr treated tumors compared to both vMyx-tdTr and PBS treatments (* - p<0.05).

We next tested whether this new recombinant virus would affect cellular immune responses *in vivo*. Because NK cells are responsive to IL15 [36], we investigated whether treatment with vMyx-IL15Rα-tdTr was associated with the presence of NK cells in subcutaneous tumors of RAG1^-/-^ mice, which have NK cells but no T or B cells. Accordingly, RAG1^-/-^ mice bearing established subcutaneous B16-F10 tumors were injected intratumorally (i.t.) with vMyx-IL15Rα-tdTr, vMyx-tdTr or PBS on days 7 and 10 post tumor cell injection. Tumor sections of mice treated with the virus expressing IL15Rα-IL15 fusion protein showed dramatic and significant increase in numbers of infiltrating NK cells, compared to vMyx-tdTr and PBS treated tumor (Figure 4). This evidence suggests a role of NK cells as a component of the host immune system that may contribute to an anti-tumor effect.

### vMyx-IL15Rα-tdTr treatment enhances both NK cell and T cell recruitment to subcutaneous tumors in C57BL/6 mice

**Figure 5:**
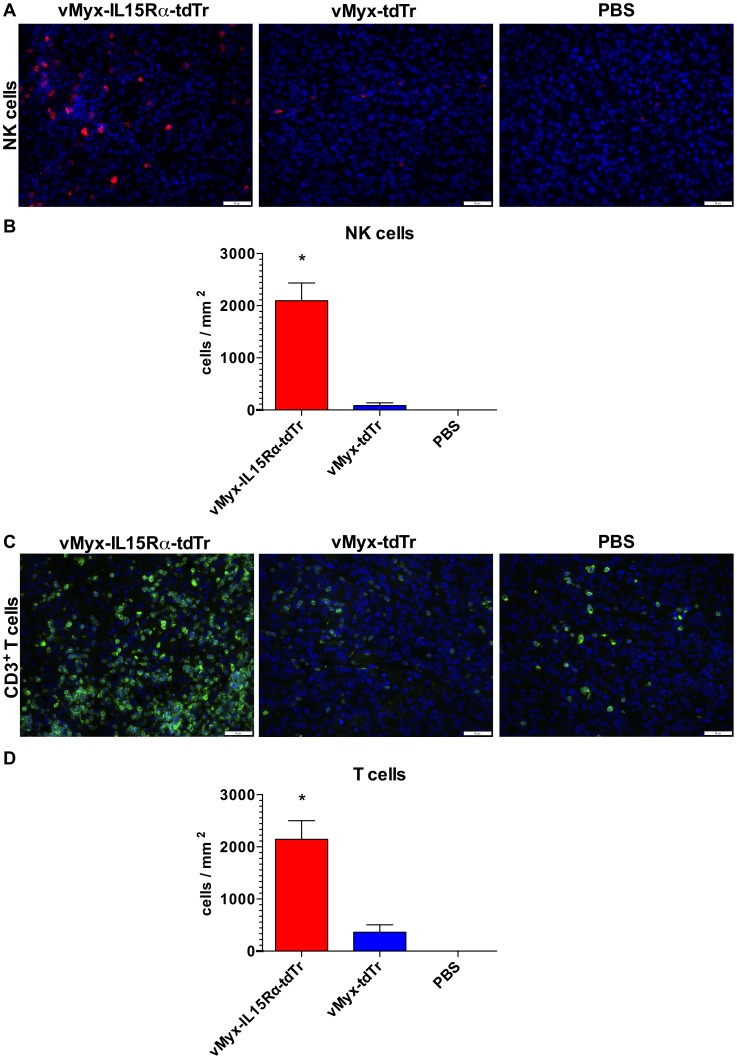
NK and T cell infiltration of subcutaneous B16-F10 tumors 3 days after intratumoral virus treatment in C57BL/6 mice. C57BL/6 mice (n = 3 per group) were implanted with unilateral subcutaneous B16-F10 tumor cells. The first dose of the virus (2.6×10^7^ PFU i.t.) was given on day 7 (when tumors reached approximately 100 mm^3^) and the second dose was given on day 10. Treatment groups are: 1. vMyx-IL15Rα-tdTr, 2. vMyx-tdTr, 3. PBS. Mice were euthanized 3 days after the final virus treatment and tumor sections were analyzed for presence of NK cells and T cells by immunostaining for Ly-49G2 (4D11 antibody) and CD3, respectively. Representative tumor sections are shown. (A) Staining for NK cells in tumors. Red – 4D11-positive stain, Blue – DAPI. Scale bar  = 50 micrometers. (B) Estimated number of NK cells per square millimeter of a tumor section for each condition, mean values and SEM from 3 mice per group. One-way ANOVA showed significant increase in NK cell accumulation in vMyx-IL15Rα-tdTr treated tumors compared to both vMyx-tdTr and PBS treatments (* - p <0.05). (C) Staining for T cells in tumors. Green – CD3-positive cells, Blue – DAPI. Scale bar  = 50 micrometers. (C) Estimated number of T cells per square millimeter of a tumor section for each condition, mean values and SEM from 3 mice per group. One-way ANOVA showed significant increase in T cell accumulation in vMyx-IL15Rα-tdTr treated tumors compared to both vMyx-tdTr and PBS treatments (* - p<0.05).

**Figure 6:**
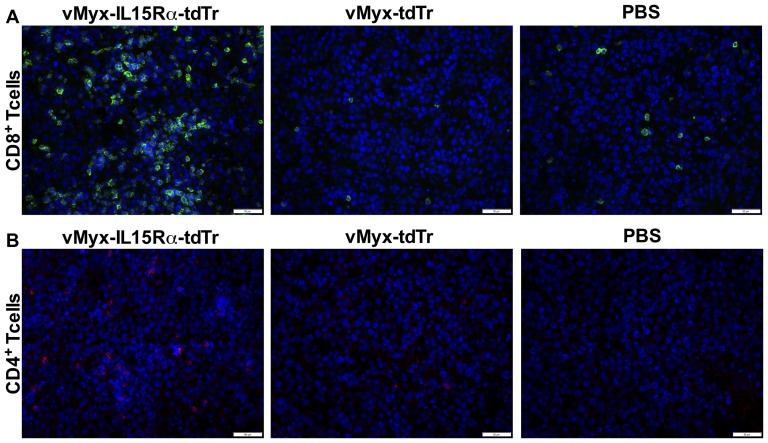
Analysis of subsets of T cells infiltrating subcutaneous B16-F10 tumors 3 days after intratumoral virus treatment in C57BL/6 mice. C57BL/6 mice (n = 3 per group) were implanted with unilateral subcutaneous B16-F10 tumor cells. The first dose of the virus (2.6×10^7^ PFU i.t.) was given on day 7 (when tumors reached approximately 100 mm^3^) and the second dose was given on day 10. Treatment groups are: 1. vMyx-IL15Rα-tdTr, 2. vMyx-tdTr, 3. PBS. Mice were euthanized 3 days after the final virus treatment and tumor sections were analyzed for presence of T cells by immunostaining for CD4 and CD8 markers. Representative tumor sections are shown. (A) Staining for CD8^+^ T cells in tumors. Green – CD8-positive cells, Blue – DAPI. Scale bar  = 50 micrometers. (B) Staining for CD4^+^ T cells in tumors. Red – CD4-positive cells, Blue – DAPI. Scale bar  = 50 micrometers.

To determine the effects of the virus in fully immunocompetent animals, we repeated the experiment using C57BL/6 mice. C57BL/6 mice with subcutaneous B16-F10 tumors were injected intratumorally with vMyx-IL15Rα-tdTr, vMyx-tdTr or PBS on days 7 and 10 post tumor cell injection. Similar to the effect observed in RAG1^-/-^ mice, C57BL/6 mice treated with vMyx-IL15Rα-tdTr also had significant intra-tumor infiltration of NK cells, compared to both tdTomato expressing virus and PBS treatment (Figure 5A, B). In addition, T cell infiltration mirrored that of NK cells (Figure 5C, D). Analysis of T cell subsets in this response revealed that most tumor infiltrating T cells were CD8^+^, although CD4^+^ cells were also elevated in vMyx-IL15Rα-tdTr treated tumors compared to controls (Figure 6).

### Mice bearing subcutaneous melanoma tumors live longer when treated with IL15Rα-IL15 fusion protein-expressing virus compared to control viruses

**Figure 7:**
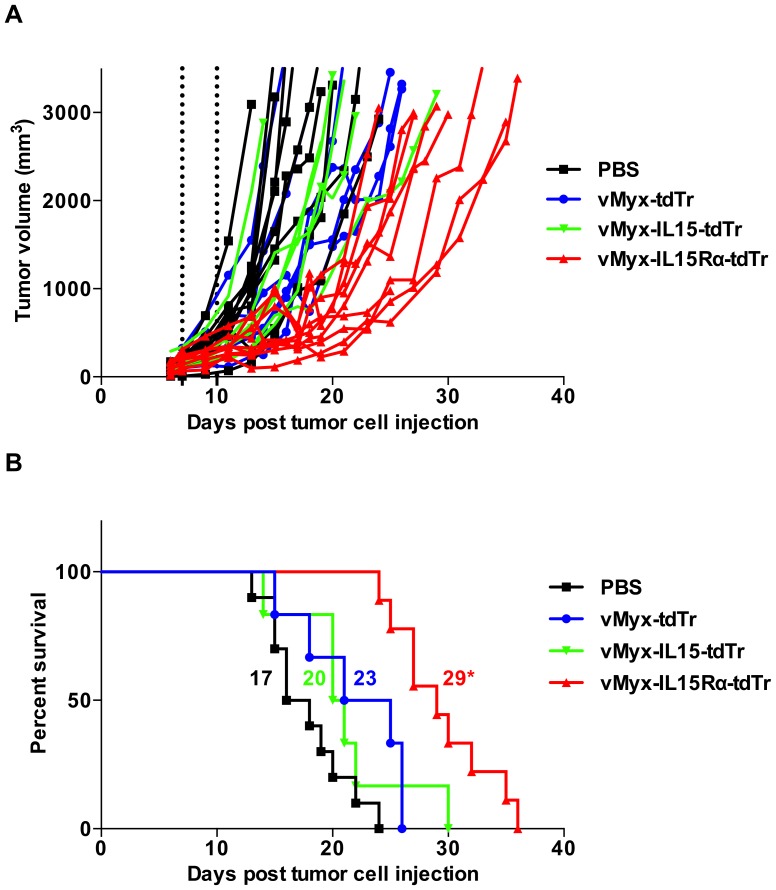
Prolonged survival of RAG1^-/-^ mice bearing subcutaneous B16-F10 tumors treated with vMyx-IL15Rα-tdTr intratumorally. RAG1^-/-^ mice (10 mice in vMyx-IL15Rα-tdTr and PBS and 6 mice in vMyx-IL15-tdTr and vMyx-tdTr treatment groups) were implanted with subcutaneous B16-F10 tumor cells. 7 days later, when tumors reached approximately 100 mm^3^, virus was inoculated intratumorally (i.t.) with 2.6×10^7^ PFU vMyx-IL15Rα-tdTr, vMyx-IL15-tdTr, vMyx-tdTr or PBS. Mice received a second i.t. inoculation of 2.6×10^7^ PFU of each virus on day 10. (A) Growth of individual tumors. Dashed lines designate time of virus treatment, and growth of tumors was measured every 2 days. (B) Kaplan–Meier survival curve of the same experimental subjects. Numbers next to corresponding survival curves designate median survival time (days). (* - p<0.05 for vMyx-IL15Rα-tdTr treated group compared to other vMyx, as well as PBS treatment).

**Figure 8:**
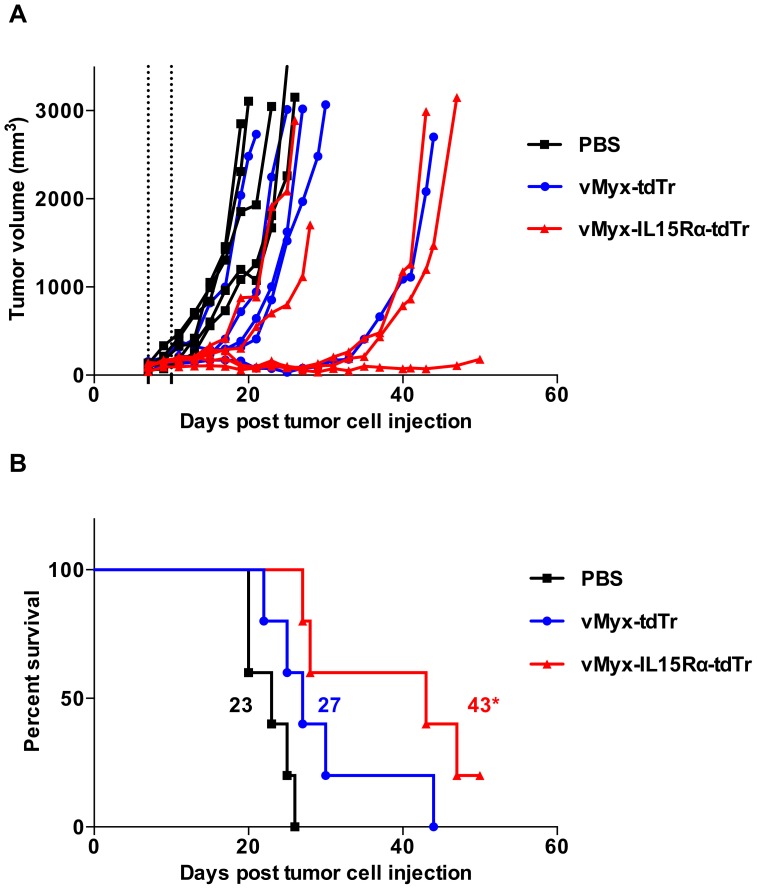
Prolonged survival of C57BL/6 mice bearing subcutaneous B16-F10 tumors treated with vMyx-IL15Rα-tdTr intratumorally. C57BL/6 mice (5 mice per group) were implanted with subcutaneous B16-F10 tumor cells. 7 days later, when tumors reached approximately 100 mm^3^, virus was inoculated intratumorally (i.t.) with 2.6×10^7^ PFU vMyx-IL15Rα-tdTr, vMyx-tdTr or PBS. Mice received a second i.t. inoculation of 2.6×10^7^ PFU of each virus on day 10. (A) Growth of individual tumors. Dashed lines designate time of virus treatment, and tumor size was measured every 2 days. (B) Kaplan–Meier survival curve of the same experimental subjects. Numbers next to corresponding survival curves designate median survival time (days). (* - p<0.05 for vMyx-IL15Rα-tdTr treated group compared to PBS).

For survival experiments, mice with established B16-F10 s.c. tumors were treated the same way as described for histological analysis (intratumoral virus treatment on days 7 and 10 post tumor cell injection) and were monitored for survival. For RAG1^-/-^ mice, treatment groups were vMyx-IL15Rα-tdTr, vMyx-IL15-tdTr, vMyx-tdTr and PBS. Without any treatment (PBS), B16-F10 grows as an exceptionally aggressive tumor, with a median survival of 17 days. A small survival benefit was observed in the tdTomato-only expressing virus group, similar to values obtained in a slightly different experimental setting [30]. Addition of the IL15 alone to the virus construct did not result in any improvement above this survival in RAG1^-/-^ mice. However, addition of the IL15Rα-IL15 fusion protein improved therapeutic efficacy of myxoma virus compared to the other virus controls, including myxoma virus that expressed only the native IL15 domain (Figure 7). vMyx-IL15Rα-tdTr treatment resulted in tumor stabilization in the majority of animals until day 20, while mice given other treatments were succumbing to tumors at this point (Figure 7A). For C57BL/6 mice, treatment groups were vMyx-IL15Rα-tdTr, vMyx-tdTr and PBS. The anti-tumor effect of vMyx-IL15Rα-tdTr in immunocompetent animals showed the same pattern as in RAG1^-/-^ mice, with overall longer median survival in corresponding groups (Figure 8). The effect on both strains is especially notable given that the time of treatment was when the tumors were already established and at the start of their aggressive growth phase.

## Discussion

IL15 has been proposed as a useful cytokine for immunotherapy for cancer, and the complexing of IL15 with its receptor alpha component has been shown to enhance its biological activity. We therefore modified a viral system to deliver the fusion protein of IL15Rα-IL15, employing a myxoma virus vector with a strong safety profile. We confirmed that the vMyx-IL15Rα-tdTr virus has the same ability to infect melanoma cells as the previously characterized vMyx-tdTr control virus, and that it secretes biologically active IL15Rα-IL15.

IL15Rα-IL15 could potentially be delivered to tumors by a variety of means. For example, Bessard et al. delivered an IL15Rα-IL15 fusion protein by three systemic injections, prolonging the survival with a B16-F10 model for 7 days [28], and Dubois et al. injected IL15 preassociated with IL15RαFc, repeated as many as nine times, prolonging survival of B16-F10 bearing mice for 5 days [24]. In comparison, in the present study two injections of vMyx-IL15Rα-tdTr resulted in a prolongation of survival of 12 days in RAG1^-/-^ mice and 20 days in C57BL/6 mice. Delivery by a viral vector results in secretion of virally encoded proteins peaking at 48–72 h and persisting for up to a week [31], so most likely fewer treatments would be needed to maintain the presence of the cytokine in the tumor environment.

In the survival experiments in RAG1^-/-^ mice we compared effects of vMyx-IL15Rα-tdTr with vMyx-IL15-tdTr [29] (virus expressing IL15 but without the IL15Rα fusion component) as well as non-cytokine expressing vMyx-tdTr. Based on recent literature [23],[37],[38],[39],[40], adding the IL15Rα significantly improves IL15 effects compared to the cytokine itself. This was confirmed in our experimental setting: IL15-only expressing virus, consistent with published data [30], showed therapeutic effect against murine melanoma tumors in the RAG1-knockout background indistinguishable from vMyx-tdTr. Hence, for most of our other studies we compared the novel recombinant virus with the variant that was closer to wild type, expressing only the fluorescent protein.

Both NK cells and CD8^+^ T cells responded to vMyx-IL15Rα-tdTr. In RAG1^-/-^ mice, histological analysis revealed robust NK cell accumulation in the tumors of the treated animals. Previous studies have done depletion of NK cells prior to treatment to show that NK cells contribute to the anti-tumor effect of IL15/IL15Rα [28],[41]. In some models, the effect of IL15Rα-IL15 is more dependent on CD8^+^ T cells [27]. In immunocompetent C57BL/6 mice, both NK cells and CD8^+^ T cells heavily infiltrated the tumors following vMyx-IL15Rα-tdTr treatment. Consistent with the idea that both cell types play a role in the effects of vMyx-IL15Rα-tdTr, treated C57BL/6 mice survived longer than treated RAG1^-/-^ mice (43 days versus 29 days, p<0.05). In our previous report of the effects of vMyx-IL15-tdTr without the receptor-α [30], we observed a significant increase in the number of CD3^+^ cells but no significant increase in survival. Quantitation of cell numbers was conducted differently in the two studies, but the density of infiltration by CD3^+^ cells following vMyx-IL15Rα-tdTr treatment appears greater than following vMyx-IL15-tdTr treatment, consistent with numerous reports of greater biological activity of IL15 when it is combined with its receptor-α subunit.

Elpek et al. observed that sustained activation of NK cells by IL15/IL15Rα treatment (5 injections over 2 weeks) can lead to functional exhaustion of effector functions of NK cells [42]. Viral delivery by myxoma virus produces IL15Rα-IL15 secretion that is intermediate between a rapidly cleared systemic injection and chronic exposure. Future studies could determine an optimal interval for repetitive treatments to minimize NK cell exhaustion. In order to assess the effector function of infiltrating NK cells and T cells, we attempted to dual-stain tumor sections with anti-interferon-γ and markers for NK cells or T cells. However, consistent with a report by Van der Loos [43] that interferon-γ is lost from sectioned tissue, we did not observe interferon-γ staining in the tumor, lymph nodes, or spleen.

In addition to the delivery of IL15Rα-IL15, the myxoma construct itself may contribute to an enhanced immune response. Previously we demonstrated the feasibility of combining adoptive T cell therapy with concurrent administration of an oncolytic virus [31]. There are at least three potential mechanisms by which myxoma virus could kill susceptible tumor cells: First, virus can directly kill tumor cells by viral oncolysis; second, local production of anti-tumor cytokines caused by viral infection can lead to recruitment and activation of immune cells that better recognize and kill tumor cells; third, killed cancer cells can be a more potent source of cross-presented tumor peptides by tumor stroma to further enhance the acquired anti-tumor immune response [44]. Manipulation of tumor microenvironment is an important strategy to improve adoptive T cell therapy and eliminate occurrence of antigen loss variants (ALV), cells that lose the T cell reactive epitope and eventually lead to tumor outgrowth [31]. We hypothesized that delivery of a highly functional and potent IL15Rα-IL15 cytokine, especially in the context of viral infection, would provide a necessary boost to immune cells in driving their functional anti-tumor activities. Potential combination therapy along with the immunomodulating activities of anti-PD1/PDL1 antibodies, might provide an even more robust initial response and elimination of ALVs [45],[46],[27].

In summary, the use of delivery systems such as vMyx-IL15Rα-tdTr, and related genetically modified viruses, has the potential to improve clinical outcomes of cancer therapy.

## Supporting Information

Figure S1 **Recombinant plasmid for modifying WT myxoma virus and generating vMyx-IL15Rα-tdTr.** Plasmid pBS-IL15Rα-IL15-tdTomatoRed (6267bp) is based on the pBluescript backbone on which M135 and M136 partial viral gene sequences are flanking genes for IL15Rα-IL15 fusion protein and tdTomato red fluorescent protein, both under control of vvSynE/L viral promoters. This expression cassette is flanked by partial viral gene sequences for the purpose of being transfected into the WT vMyx-Lau virus genome between genes M135 and M136.
(TIFF)

Figure S2 **Western blot showing presence of IL15Rα-IL15 fusion protein in the supernatants of cells infected with vMyx-IL15Rα-tdTr.** Confluent RK-13 cells in 6-well plates were infected with vMyx-IL15Rα-tdTr or vMyx-tdTr at MOI = 5. Cell media was collected and cells were scraped, lysed and cytoplasmic extract was harvested at 48 h post-infection. Membranes blotted with supernatants and cell extracts of virus infected cells were stained for IL15 (left panel) or IL15Rα (right panel). Experiment was repeated five times with similar results. (SNT – supernatant, CE – cell extract).
(TIFF)

Materials and Methods S1 **Western blot analysis.**
(DOCX)

## References

[1] Russell SJ, Peng K-W, Bell JC (2012) Oncolytic virotherapy. Nat Biotechnol 30: 658–670 10.1038/nbt.2287 22781695 PMC3888062

[2] Stephenson KB, Barra NG, Davies E, Ashkar AA, Lichty BD (2011) Expressing human interleukin-15 from oncolytic vesicular stomatitis virus improves survival in a murine metastatic colon adenocarcinoma model through the enhancement of anti-tumor immunity. Cancer Gene Ther 19: 238–246 10.1038/cgt.2011.81 22158521

[3] Lun X, Chan J, Zhou H, Sun B, Kelly JJP, et al (2010) Efficacy and safety/toxicity study of recombinant vaccinia virus JX-594 in two immunocompetent animal models of glioma. Mol Ther J Am Soc Gene Ther 18: 1927–1936 10.1038/mt.2010.183 PMC2990519 20808290

[4] Chan WM, Rahman MM, McFadden G (2013) Oncolytic myxoma virus: the path to clinic. Vaccine 31: 4252–4258 10.1016/j.vaccine.2013.05.056 23726825 PMC3755036

[5] Sypula J, Wang F, Ma Y, Bell J (2004) McFadden G (2004) Myxoma virus tropism in human tumor cells. Gene Ther Mol Biol 8: 103–114.

[6] Barrett JW, Alston LR, Wang F, Stanford MM, Gilbert P-A, et al (2007) Identification of host range mutants of myxoma virus with altered oncolytic potential in human glioma cells. J Neurovirol 13: 549–560 10.1080/13550280701591526 18097886

[7] Wang G, Barrett JW, Stanford M, Werden SJ, Johnston JB, et al (2006) Infection of human cancer cells with myxoma virus requires Akt activation via interaction with a viral ankyrin-repeat host range factor. Proc Natl Acad Sci U S A 103: 4640–4645 10.1073/pnas.0509341103 16537421 PMC1450224

[8] Lun X, Yang W, Alain T, Shi Z-Q, Muzik H, et al (2005) Myxoma Virus Is a Novel Oncolytic Virus with Significant Antitumor Activity against Experimental Human Gliomas. Cancer Res 65: 9982–9990 10.1158/0008-5472.CAN-05-1201 16267023 PMC4373463

[9] Lun XQ, Zhou H, Alain T, Sun B, Wang L, et al (2007) Targeting human medulloblastoma: oncolytic virotherapy with myxoma virus is enhanced by rapamycin. Cancer Res 67: 8818–8827 10.1158/0008-5472.CAN-07-1214 17875723 PMC4380180

[10] Wu Y, Lun X, Zhou H, Wang L, Sun B, et al (2008) Oncolytic efficacy of recombinant vesicular stomatitis virus and myxoma virus in experimental models of rhabdoid tumors. Clin Cancer Res Off J Am Assoc Cancer Res 14: 1218–1227 10.1158/1078-0432.CCR-07-1330 PMC2844789 18281557

[11] Wennier ST, Liu J, Li S, Rahman MM, Mona M, et al (2012) Myxoma Virus Sensitizes Cancer Cells to Gemcitabine and Is an Effective Oncolytic Virotherapeutic in Models of Disseminated Pancreatic Cancer. Mol Ther 20: 759–768 10.1038/mt.2011.293 22233582 PMC3321583

[12] Stanford MM, Shaban M, Barrett JW, Werden SJ, Gilbert P-A, et al (2008) Myxoma virus oncolysis of primary and metastatic B16F10 mouse tumors in vivo. Mol Ther J Am Soc Gene Ther 16: 52–59 10.1038/sj.mt.6300348 PMC4376281 17998900

[13] Lun X, Alain T, Zemp FJ, Zhou H, Rahman MM, et al (2010) Myxoma Virus Virotherapy for Glioma in Immunocompetent Animal Models: Optimizing Administration Routes and Synergy with Rapamycin. Cancer Res 70: 598–608 10.1158/0008-5472.CAN-09-1510 20068158

[14] Bartee E, Chan WM, Moreb JS, Cogle CR, McFadden G (2012) Selective purging of human multiple myeloma cells from autologous stem cell transplantation grafts using oncolytic myxoma virus. Biol Blood Marrow Transplant J Am Soc Blood Marrow Transplant 18: 1540–1551 10.1016/j.bbmt.2012.04.004 PMC3406238 22516053

[15] Rahman MM, Madlambayan GJ, Cogle CR, McFadden G (2010) Oncolytic viral purging of leukemic hematopoietic stem and progenitor cells with Myxoma virus. Cytokine Growth Factor Rev 21: 169–175 10.1016/j.cytogfr.2010.02.010 20211576 PMC2881168

[16] Senzer NN, Kaufman HL, Amatruda T, Nemunaitis M, Reid T, et al (2009) Phase II clinical trial of a granulocyte-macrophage colony-stimulating factor-encoding, second-generation oncolytic herpesvirus in patients with unresectable metastatic melanoma. J Clin Oncol Off J Am Soc Clin Oncol 27: 5763–5771 10.1200/JCO.2009.24.3675 19884534

[17] Heo J, Reid T, Ruo L, Breitbach CJ, Rose S, et al (2013) Randomized dose-finding clinical trial of oncolytic immunotherapeutic vaccinia JX-594 in liver cancer. Nat Med 10.1038/nm.3089 PMC4268543 23396206

[18] Parmiani G, Castelli C, Pilla L, Santinami M, Colombo MP, et al (2007) Opposite immune functions of GM-CSF administered as vaccine adjuvant in cancer patients. Ann Oncol Off J Eur Soc Med Oncol ESMO 18: 226–232 10.1093/annonc/mdl158 17116643

[19] Cawood R, Hills T, Wong SL, Alamoudi AA, Beadle S, et al (2012) Recombinant viral vaccines for cancer. Trends Mol Med 18: 564–574 10.1016/j.molmed.2012.07.007 22917663

[20] Cheever MA (2008) Twelve immunotherapy drugs that could cure cancers. Immunol Rev 222: 357–368 10.1111/j.1600-065X.2008.00604.x 18364014

[21] Steel JC, Waldmann TA, Morris JC (2012) Interleukin-15 biology and its therapeutic implications in cancer. Trends Pharmacol Sci 33: 35–41 10.1016/j.tips.2011.09.004 22032984 PMC3327885

[22] Dubois S, Mariner J, Waldmann TA, Tagaya Y (2002) IL-15Rα Recycles and Presents IL-15 In trans to Neighboring Cells. Immunity 17: 537–547 10.1016/S1074-7613(02)00429-6 12433361

[23] Stoklasek TA, Schluns KS, Lefrançois L (2006) Combined IL-15/IL-15Rα Immunotherapy Maximizes IL-15 Activity In Vivo. J Immunol 177: 6072–6080. 17056533 10.4049/jimmunol.177.9.6072 PMC2847275

[24] Dubois S, Patel HJ, Zhang M, Waldmann TA, Müller JR (2008) Preassociation of IL-15 with IL-15Rα-IgG1-Fc Enhances Its Activity on Proliferation of NK and CD8+/CD44high T Cells and Its Antitumor Action. J Immunol 180: 2099–2106. 18250415 10.4049/jimmunol.180.4.2099

[25] Rubinstein MP, Kovar M, Purton JF, Cho J-H, Boyman O, et al (2006) Converting IL-15 to a superagonist by binding to soluble IL-15R{alpha}. Proc Natl Acad Sci U S A 103: 9166–9171 10.1073/pnas.0600240103 16757567 PMC1482584

[26] Jakobisiak M, Golab J, Lasek W (2011) Interleukin 15 as a promising candidate for tumor immunotherapy. Cytokine Growth Factor Rev 22: 99–108 10.1016/j.cytogfr.2011.04.001 21531164

[27] Epardaud M, Elpek KG, Rubinstein MP, Yonekura A, Bellemare-Pelletier A, et al (2008) Interleukin-15/interleukin-15R alpha complexes promote destruction of established tumors by reviving tumor-resident CD8+ T cells. Cancer Res 68: 2972–2983 10.1158/0008-5472.CAN-08-0045 18413767

[28] Bessard A, Solé V, Bouchaud G, Quéméner A, Jacques Y (2009) High antitumor activity of RLI, an interleukin-15 (IL-15)-IL-15 receptor alpha fusion protein, in metastatic melanoma and colorectal cancer. Mol Cancer Ther 8: 2736–2745 10.1158/1535-7163.MCT-09-0275 19723883

[29] Liu J, Wennier S, Reinhard M, Roy E, MacNeill A, et al (2009) Myxoma virus expressing interleukin-15 fails to cause lethal myxomatosis in European rabbits. J Virol 83: 5933–5938 10.1128/JVI.00204-09 19279088 PMC2681933

[30] MacNeill AL, Doty RA, Liu J, McFadden G, Roy EJ (2013) Histological evaluation of intratumoral myxoma virus treatment in an immunocompetent mouse model of melanoma. Oncolytic Virotherapy 1 10.2147/OV.S37971 PMC4389688 25866742

[31] Thomas DL, Doty R, Tosic V, Liu J, Kranz DM, et al (2011) Myxoma virus combined with rapamycin treatment enhances adoptive T cell therapy for murine melanoma brain tumors. Cancer Immunol Immunother CII 60: 1461–1472 10.1007/s00262-011-1045-z 21656158 PMC4378695

[32] Stone JD, Chervin AS, Schreiber H, Kranz DM (2012) Design and characterization of a protein superagonist of IL-15 fused with IL-15Rα and a high-affinity T cell receptor. Biotechnol Prog 28: 1588–1597 10.1002/btpr.1631 22961781 PMC3514595

[33] Spiotto MT, Yu P, Rowley DA, Nishimura MI, Meredith SC, et al (2002) Increasing tumor antigen expression overcomes “ignorance” to solid tumors via crosspresentation by bone marrow-derived stromal cells. Immunity 17: 737–747. 12479820 10.1016/s1074-7613(02)00480-6

[34] Blank C, Brown I, Peterson AC, Spiotto M, Iwai Y, et al (2004) PD-L1/B7H-1 Inhibits the Effector Phase of Tumor Rejection by T Cell Receptor (TCR) Transgenic CD8+ T Cells. Cancer Res 64: 1140–1145 10.1158/0008-5472.CAN-03-3259 14871849

[35] Kaspar M, Trachsel E, Neri D (2007) The Antibody-Mediated Targeted Delivery of Interleukin-15 and GM-CSF to the Tumor Neovasculature Inhibits Tumor Growth and Metastasis. Cancer Res 67: 4940–4948 10.1158/0008-5472.CAN-07-0283 17510424

[36] Marçais A, Viel S, Grau M, Henry T, Marvel J, et al (2013) Regulation of Mouse NK Cell Development and Function by Cytokines. Front Immunol 4: 450 10.3389/fimmu.2013.00450 24376448 PMC3859915

[37] Castillo EF, Schluns KS (2012) Regulating the immune system via IL-15 transpresentation. Cytokine 59: 479–490 10.1016/j.cyto.2012.06.017 22795955 PMC3422378

[38] Kermer V, Baum V, Hornig N, Kontermann RE, Müller D (2012) An Antibody Fusion Protein for Cancer Immunotherapy Mimicking IL-15 trans-Presentation at the Tumor Site. Mol Cancer Ther 11: 1279–1288 10.1158/1535-7163.MCT-12-0019 22491823

[39] Bouchaud G, Garrigue-Antar L, Solé V, Quéméner A, Boublik Y, et al (2008) The exon-3-encoded domain of IL-15ralpha contributes to IL-15 high-affinity binding and is crucial for the IL-15 antagonistic effect of soluble IL-15Ralpha. J Mol Biol 382: 1–12 10.1016/j.jmb.2008.07.019 18656487

[40] Liu RB, Engels B, Arina A, Schreiber K, Hyjek E, et al (2012) Densely Granulated Murine NK Cells Eradicate Large Solid Tumors. Cancer Res 72: 1964–1974 10.1158/0008-5472.CAN-11-3208 22374983 PMC3680344

[41] Rowley J, Monie A, Hung C-F, Wu T-C (2008) Inhibition of Tumor Growth by NK1.1+ Cells and CD8+ T Cells Activated by IL-15 through Receptor β/Common γ Signaling in trans. J Immunol 181: 8237–8247. 19050240 10.4049/jimmunol.181.12.8237 PMC3071607

[42] Elpek KG, Rubinstein MP, Bellemare-Pelletier A, Goldrath AW, Turley SJ (2010) Mature natural killer cells with phenotypic and functional alterations accumulate upon sustained stimulation with IL-15/IL-15Rα complexes. Proc Natl Acad Sci 107: 21647–21652 10.1073/pnas.1012128107 21098276 PMC3003106

[43] Van der Loos CM, Houtkamp MA, de Boer OJ, Teeling P, van der Wal AC, et al (2001) Immunohistochemical detection of interferon-gamma: fake or fact? J Histochem Cytochem Off J Histochem Soc 49: 699–710. 10.1177/002215540104900604 11373317

[44] Zhang B, Bowerman NA, Salama JK, Schmidt H, Spiotto MT, et al (2007) Induced sensitization of tumor stroma leads to eradication of established cancer by T cells. J Exp Med 204: 49–55 10.1084/jem.20062056 17210731 PMC2118433

[45] Pardoll DM (2012) The blockade of immune checkpoints in cancer immunotherapy. Nat Rev Cancer 12: 252–264 10.1038/nrc3239 22437870 PMC4856023

[46] Xu W, Jones M, Liu B, Zhu X, Johnson CB, et al (2013) Efficacy and Mechanism-of-Action of a Novel Superagonist Interleukin-15: Interleukin-15 Receptor αSu/Fc Fusion Complex in Syngeneic Murine Models of Multiple Myeloma. Cancer Res 73: 3075–3086 10.1158/0008-5472.CAN-12-2357 23644531 PMC3914673

