# Supplementary figures and images for: Myxoma Virus Expressing a Fusion Protein of Interleukin-15 (IL15) and IL15 Receptor Alpha Has Enhanced Antitumor Activity
Source: PLoS One. 2014 Oct 16;9(10):e109801. doi: 10.1371/journal.pone.0109801 (PMC4199602; doi:10.1371/journal.pone.0109801)

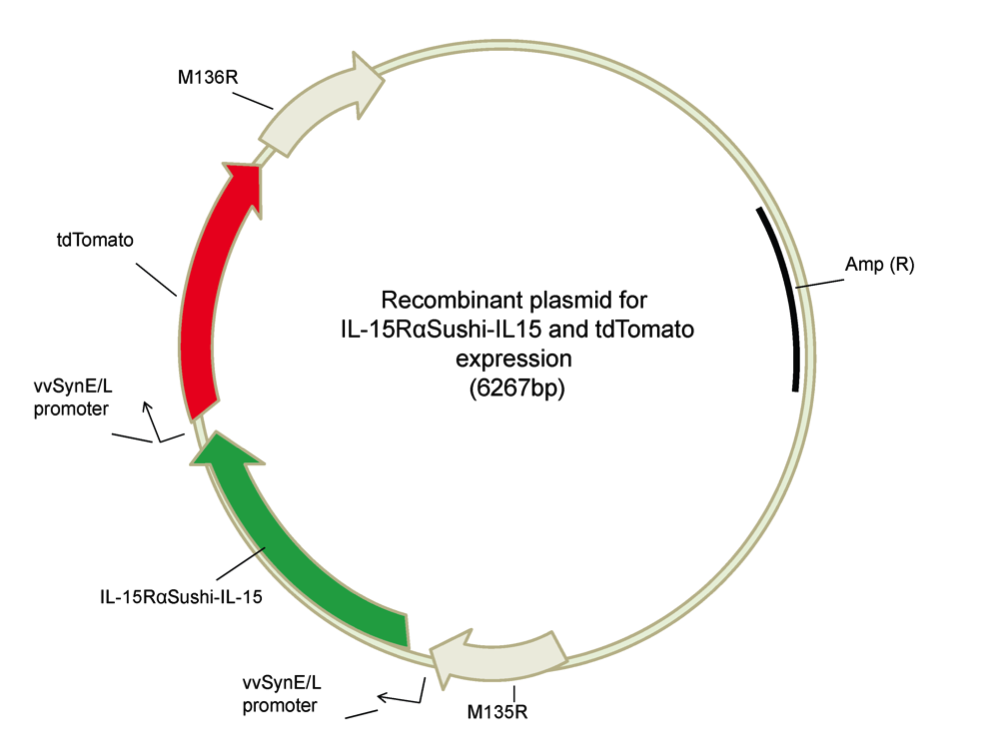

Supplement: Figure S1 — Recombinant plasmid for modifying WT myxoma virus and generating vMyx-IL15Rα-tdTr. Plasmid pBS-IL15Rα-IL15-tdTomatoRed (6267bp) is based on the pBluescript backbone on which M135 and M136 partial viral gene sequences are flanking genes for IL15Rα-IL15 fusion protein and tdTomato red fluorescent protein, both under control of vvSynE/L viral promoters. This expression cassette is flanked by partial viral gene sequences for the purpose of being transfected into the WT vMyx-Lau virus genome between genes M135 and M136. (TIFF) [file pone.0109801.s001.tiff]

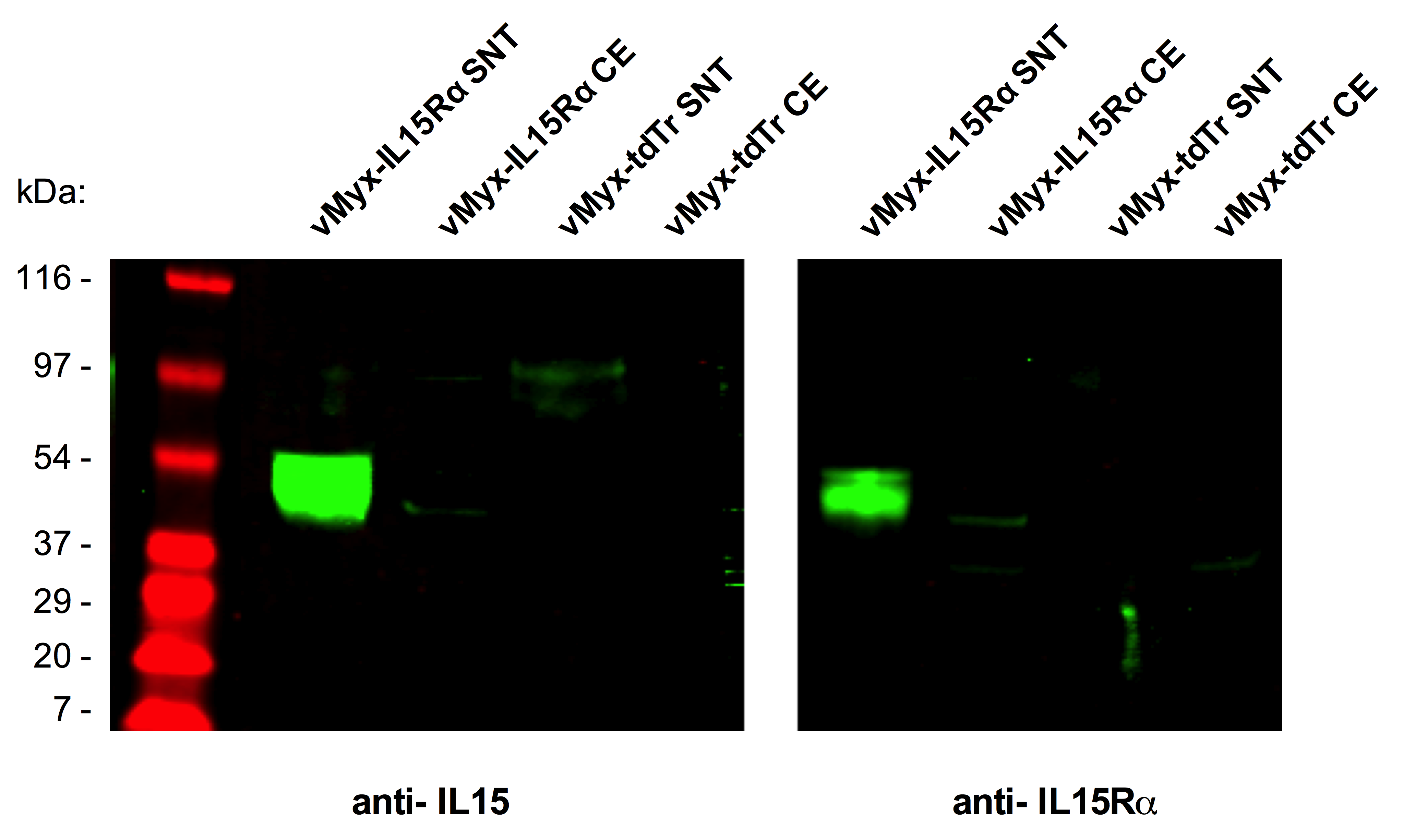

Supplement: Figure S2 — Western blot showing presence of IL15Rα-IL15 fusion protein in the supernatants of cells infected with vMyx-IL15Rα-tdTr. Confluent RK-13 cells in 6-well plates were infected with vMyx-IL15Rα-tdTr or vMyx-tdTr at MOI = 5. Cell media was collected and cells were scraped, lysed and cytoplasmic extract was harvested at 48 h post-infection. Membranes blotted with supernatants and cell extracts of virus infected cells were stained for IL15 (left panel) or IL15Rα (right panel). Experiment was repeated five times with similar results. (SNT – supernatant, CE – cell extract). (TIFF) [file pone.0109801.s002.tiff]
