# Supplementary material for: Myxoma Virus Expressing a Fusion Protein of Interleukin-15 (IL15) and IL15 Receptor Alpha Has Enhanced Antitumor Activity
Source: PLoS One. 2014 Oct 16;9(10):e109801. doi: 10.1371/journal.pone.0109801 (PMC4199602; doi:10.1371/journal.pone.0109801)
Supplement: Materials and Methods S1 — Western blot analysis. (DOCX) [file pone.0109801.s003.docx]

# Supporting Materials and Methods

## Western blot analysis

Supernatant and cell extract of virus infected cells was obtained the same way as previously described for ELISA: RK-13 cells were plated in 6-well culture plates and upon reaching 90-95% confluency they were inoculated with vMyx-IL15Rα-tdTr or vMyx-tdTr diluted in 400µL MEM-C at MOI of 5 PFU/cell. After 1h incubation at 37°C and 5% CO_2_, inoculum was replaced with MEM-C with 10% FBS. At different time points post-inoculation, both cell supernatant and cellular extract were collected. Supernatants were centrifuged briefly to remove cellular debris and clarified supernatants were transferred to new tubes and stored at -80C. The remaining cellular monolayer was detached from the well by scraping cells into 1ml PBS. Cells were collected, pelleted by brief centrifugation (1,300 rpm x 1 min), and cellular pellets were resuspended in Cytoplasmic Extract (CE) buffer supplemented with HALT protease inhibitor cocktail (Thermo Fisher, Rockford, IL). Samples were incubated for 5 min at 4°C and centrifuged at 1,300 rpm for 1 min. Supernatants were moved to new tubes are stored at -80°C.

For Western Blot analysis, samples were transferred from the SDS-PAGE gels (precast Mini-PROTEAN TGX gels by Bio-Rad, Hercules, CA) to Millipore Immobilon-P Transfer membrane (Millipore, Billerica, MA) using the semi-dry transfer method (Trans-Blot® SD Semi-Dry Transfer Cell by Bio-Rad, Hercules, CA). Membranes were blocked in 4% skim milk in PBS. Antibodies were diluted in PBST/0.5% BSA. For IL15 detection, anti-IL15-biotin antibody from eBioscience (San Diego, CA) was used, coupled with IRDye 800CW Streptavidin (SA-IRD800) from LI-COR (LI-COR Biosciences, US). For IL-15Rα detection, Mouse IL15 R alpha Affinity Purified Polyclonal Ab (R&D Systems, Minneapolis, MN) was used as a primary antibody, Rabbit polyclonal Antibody to Goat IgG - H&L (Biotin) (Abcam, Cambridge, MA) was used as a secondary antibody and finalized with SA-IRD800 as described previously. Blots were visualized on the Odyssey Infrared Imaging System (LI-COR Biosciences, US). Molecular weight markers used were Broad Range Prestained SDS-PAGE Standards from Bio-Rad (Hercules, CA). In each repeat of the experiment two separate, identically loaded SDS-PAGE gels were run, samples from each gel were transferred to a corresponding membrane, one of which was stained for IL15, and the other for IL15Rα.
